# Associations Between Endocrine Status and Stress, Mood and Psychosomatic Status in Elite Handball Players

**DOI:** 10.3390/sports14070289

**Published:** 2026-07-08

**Authors:** Fanny Zselyke Ratz-Sulyok, Csilla Jang-Kapuy, Peter Bakonyi, Bettina Beres, Tamas Dobronyi, Gergo Simon, Annamaria Zsakai, Tamas Szabo

**Affiliations:** 1 Sport Sciences and Diagnostic Research Centre, Hungarian Handball Federation, 1103 Budapest, Hungary; rsfzs5@gmail.com (F.Z.R.-S.); kapuycsilla@gmail.com (C.J.-K.); bakonyi.peter@mksz.hu (P.B.); beres.bettina@mksz.hu (B.B.); dobronyi.tamas@mksz.hu (T.D.); simon.gergo@mksz.hu (G.S.); szabo.tamas@mksz.hu (T.S.); 2 Department of Biological Anthropology, Eotvos Lorand University, ELTE, 1117 Budapest, Hungary; 3 Doctoral School of Biology, Eotvos Lorand University, ELTE, 1117 Budapest, Hungary

**Keywords:** salivary hormone levels, stress status, mood profile, psychosomatic symptoms, elite athletes

## Abstract

Purpose: The assessment of endocrine status in elite athletes is typically linked to training load and perceived stress; however, the relationship between hormonal parameters and psychosomatic and stress indicators remains insufficiently understood. This study aimed to investigate the associations between endocrine status and stress, mood, and psychosomatic status indicators in elite handball players. Methods: In a cross-sectional study, salivary cortisol (with no strict control over wake-up time), testosterone, and—in female athletes—17-β-estradiol concentrations were assessed in 584 elite handball players aged 14–35 years using ELISA. Psychological variables were evaluated using the Perceived Stress Scale (PSS), Profile of Mood States (POMS), and the Health Behavior in School-aged Children Symptom Checklist (HBSC-SCL). Associations were examined using non-parametric tests and general linear models adjusted for age. Results: Hormonal and psychological variables demonstrated significant age-related trends. No significant associations were observed between hormonal parameters and perceived stress or mood disturbance (values for the general linear model (GLM) were all *p* > 0.05). In contrast, psychosomatic symptom severity was significantly associated with cortisol levels in male athletes (GLM, *p* < 0.001) and testosterone levels in female athletes (GLM, *p* = 0.009). Multivariate analyses confirmed the relevance of psychosomatic symptoms and indicated interaction effects between stress-related factors. Conclusion: Psychosomatic symptoms were more closely associated with endocrine status than with perceived stress or mood disturbance in elite handball players. However, these associations were characterized by relatively small effect sizes, indicating that psychosomatic symptoms explain only a limited proportion of the variance in hormonal parameters. These findings suggest that psychosomatic indicators may provide a more sensitive reflection of physiological strain and support the use of integrated monitoring approaches combining endocrine and psychosomatic measures in elite sport. In practical terms, routine monitoring of psychosomatic symptoms alongside hormonal measures may help practitioners to identify early signs of physiological strain and support timely adjustments in training load and recovery strategies.

## 1. Introduction

Despite increasing attention to overtraining and relative energy deficiency in sport (RED-S), early identification of athletes experiencing physiological strain remains challenging. Endocrine parameters are considered important indicators of physiological regulation; however, most studies have focused on isolated hormonal parameters rather than integrated hormonal patterns [1,2,3,4]. Given the complex and multifactorial nature of training adaptation, a combined evaluation of hormonal profiles may provide a more sensitive approach for detecting early variations in physiological regulation without relying on fixed reference thresholds.

Handball is a physically demanding sport requiring the integration of strength, speed, agility, endurance, and technical skills. Training involves both aerobic and anaerobic components, repeated high-intensity efforts, rapid directional changes, and frequent physical contact, resulting in substantial cumulative physiological stress [5]. Consistent with these demands, previous studies have demonstrated that elite athletes exhibit endocrine profiles distinct from those of non-athlete populations [1,2,6].

In our previous analysis of elite handball players [7], male athletes exhibited higher resting testosterone levels, while female athletes displayed lower estradiol levels compared with age-matched non-athletes. Additionally, adult athletes showed elevated cortisol concentrations, although the previous literature has reported inconsistent findings regarding baseline cortisol levels in athletes [8,9]. These findings highlight sport-specific endocrine characteristics and the need to interpret hormonal profiles within athletic populations.

Elite athletes are exposed to substantial physical and psychological stress related to training load, competition, and performance expectations. These factors are commonly assessed using subjective tools such as perceived stress, mood state, and psychosomatic symptoms, which reflect different aspects of the athlete’s response to load and recovery [10,11]. Instruments such as the Perceived Stress Scale (PSS), the Profile of Mood States (POMS), and the Health Behaviour in School-aged Children Symptom Checklist (HBSC-SCL) are widely used to capture these responses [12,13].

Despite increasing interest in endocrine markers and psychological indicators in athlete monitoring, the interaction between physiological and psychological responses to training stress remains insufficiently understood in elite athletes. While hormonal parameters provide objective indicators of neuroendocrine regulation, psychological and psychosomatic measures reflect the subjective and functional consequences of training load and recovery status [14,15]. However, these domains have typically been investigated separately, and little is known about whether subjective experiences of stress and somatic complaints correspond to underlying endocrine variations in elite athlete populations [16]. Clarifying this relationship is essential, as it links biological processes with functional outcomes and may improve the sensitivity of athlete monitoring approaches [17,18,19]. While a wide range of athlete-monitoring tools exist (e.g., wellness questionnaires, recovery scales and training load metrics), these approaches primarily capture perceived or external aspects of load. In contrast, psychosomatic symptoms integrate somatic, psychological and sleep-related responses and may therefore provide a closer reflection of underlying physiological strain.

Based on these considerations, the aim of the present study was to examine the associations between endocrine status and psychological (perceived stress and mood) and psychosomatic indicators in elite handball players. We hypothesized that psychosomatic symptoms would show stronger associations with hormonal parameters than with perceived stress or mood disturbance, reflecting the distinction between perceived stress and underlying biological processes.

## 2. Materials and Methods

### 2.1. Studied Sample

A total of 584 elite adolescent and adult handball players (aged 14–35 years, Table 1) were examined between 2024 and 2025 at the Human Biology Laboratory of the Hungarian Handball Federation’s Sport Sciences and Diagnostics Research Centre. Participants were members of age-group national teams (U17, U19, U21, and adults) or were selected through a talent identification program for the U15 age group.

Athletes with known diseases or those taking medication were excluded from the analysis. Among female athletes, only those who had not used hormonal contraceptives or other exogenous sex hormones within the previous 12 months were included in the saliva sampling.

### 2.2. Sample Size Estimation and Justification

The size of the target population was inherently limited to members of the Hungarian Handball Federation’s national teams and youth players selected through a talent identification program. The potential pool of participants per season comprised approximately 370 athletes across both sexes (U15 talent program: 80 players—males: 14.8 ± 0.5 ys, females: 14.2 ± 0.4 ys, U17—males: 16.4 ± 0.6 ys, females: 14.2 ± 0.4 ys, U19—males: 18.1 ± 0.8 ys, females: 16.8 ± 0.8 ys, and U21—males: 20.0 + 1.0 ys, females: 18.8 ± 0.8 ys; 25 players each; adult—males: 28.3 ± 4.3 ys, females: 25.9 ± 4.5 ys: 30 players per sex). Each athlete was examined only once; repeated measurements were not included in the present analysis. Athletes who were ill or injured at the time of data collection were excluded.

Given the need for subgroup analyses (including sex-specific groups and, in females, stratification by menstrual cycle phase), a single-season sample was insufficient. Therefore, data collection was extended across multiple seasons to ensure adequate representation within subgroups.

A formal a priori sample size calculation was performed to estimate the minimum number of participants required for detecting meaningful differences in hormonal parameters. Assuming a medium effect size (Cohen’s d = 0.5), a statistical power of 80%, and a significance level of 0.05, the required sample size was estimated at 50–70 participants per group for primary comparisons and 40–60 participants per subgroup in sex-stratified analyses. The final sample size met these minimum requirements.

Given the restricted size of the eligible population, the study should be interpreted as a population-based assessment of elite Hungarian handball players rather than a sample drawn from a larger population.

### 2.3. Endocrine Status Assessment

The endocrine status of handball players was assessed by salivary hormone analysis. Saliva samples were collected under resting conditions using standardized procedures [20]. Cortisol and testosterone concentrations were determined for all participants, and 17-β-estradiol concentrations were additionally measured in female athletes (Table 1). Participants were instructed to abstain from eating, drinking, chewing gum, and brushing their teeth for at least 30 min prior to sampling.

Saliva samples were collected between 09:00 and 10:00 a.m. on the day of anthropometric examinations, in accordance with the recommendations of the ELISA kits (ELISA; Tecan Group Ltd., Männedorf, Switzerland). To account for diurnal variation in hormone levels, participants were instructed to wake between 06:00 and 07:00, reflecting their habitual waking time. This protocol aimed to standardize the interval between awakening and sample collection, which is particularly relevant for cortisol assessment. However, as compliance and exact awakening times could not be objectively verified, some variability in this interval may have occurred.

Salivary cortisol, testosterone, and 17-β-estradiol concentrations were determined using enzyme-linked immunosorbent assays (ELISA; Tecan Group Ltd., Männedorf, Switzerland). All standards, samples, and controls were analyzed in duplicate. Absorbance was measured at 450 nm using a Thermo Scientific Multiskan SkyHigh microplate spectrophotometer (Thermo Fisher Scientific, Waltham, MA, USA).

The ELISA kit guidelines recommend evaluating testosterone concentrations from the age of 20 years. Reference values for 17-β-estradiol are applicable from 17 years of age, with consideration of menstrual cycle phases in female participants. The determination of menstrual cycle phase was based on self-reported data obtained through personal interviews with the athletes, including information on cycle length, bleeding patterns, regularity, and the date of the last menstruation. Estradiol concentrations were expressed as a percentage of the reference median corresponding to the menstrual cycle phase in order to account for its physiological fluctuations across the cycle. Cortisol reference values are considered independent of sex and age (above 6 years), and are primarily determined by the time elapsed since awakening. Given the observed differences in cortisol distribution in preliminary analyses, the study population was divided into two age groups (below and above 18 years) for cortisol evaluation. To standardize comparisons, cortisol concentrations were expressed as a percentage of the reference median corresponding to the elapsed time since awakening (relative cortisol level).

### 2.4. Psychosomatic Health, Stress, and Mood Status Assessment

Data on psychosomatic health, perceived stress, and mood status were collected through structured personal interviews.

Perceived stress was assessed using the 14-item Perceived Stress Scale (PSS-14) [21], which measures the extent to which individuals perceive situations in their lives as stressful over the past month. Responses were rated on a 5-point Likert scale (0: never to 4: very often), with reverse scoring applied to positively worded items. Total scores were categorized into three levels: low stress (0–18), moderate stress (19–37), and high stress (38–56).

Mood states and total mood disturbance (TMD) were evaluated using the Profile of Mood States (POMS) [22]. This 65-item questionnaire assesses the intensity of various feelings experienced during the previous week on a 5-point scale (0 = not at all to 4 = extremely, with reverse scoring applied to two items). The items are grouped into six mood dimensions: tension, depression, anger, fatigue, confusion, and vigor. The TMD score was calculated by summing the negative mood components (tension, depression, anger, fatigue, confusion) and subtracting the vigor score. Based on the distribution of TMD scores within the study population, participants were classified into three categories: low mood disturbance (<25th percentile; −32 to −2), moderate mood disturbance (25–75th percentile; −2 to 29.5), and high mood disturbance (>75th percentile; 29.5 to 200).

Psychosomatic symptom frequency was assessed using the Health Behavior in School-aged Children Symptom Checklist (HBSC-SCL, 8 items) [23]. The instrument captures somatic, psychological, and sleep-related symptoms experienced over the previous six months. Responses were recorded on a 5-point scale (0: rarely or never to 4: about every day). Based on total scores, participants were classified into three risk categories: low risk (0–7, rare psychosomatic symptoms), moderate risk (8–11, average level of psychosomatic symptoms), and high risk (≥12, frequent psychosomatic symptoms).

### 2.5. Statistical Analyses

Statistical analyses were performed using IBM SPSS Statistics (version 29.0; IBM Corp., Armonk, NY, USA). The distribution of continuous variables was assessed using the Shapiro–Wilk test. As hormonal and psychological variables showed non-normal distribution, non-parametric statistical methods were applied for descriptive and exploratory analyses.

Continuous variables are presented as median, interquartile range (25–75th centiles) and range (minimum–maximum), while categorical variables are expressed as frequencies and percentages. Age-related differences in hormonal parameters and psychological indicators were examined using the Kruskal–Wallis test. Differences in the distribution of categorical variables were assessed using the chi-square (χ^2^) test where applicable.

To explore differences in hormonal parameters across categories of psychological and psychosomatic indicators (Perceived Stress Scale score—PSS, Profile of Mood States score—POMS and Health Behavior in School-aged Children Symptom Checklist—HBSC8) covering 4 somatic symptoms (headache, stomach ache, backache, dizziness), 3 psychological symptoms (feeling low, irritability, nervousness), and difficulty sleeping (as a sleep-related symptom), non-parametric subgroup comparisons were conducted using Kruskal–Wallis tests for three-group comparisons and Mann–Whitney U tests for two-group comparisons.

To examine the association between hormonal parameters and stress, mood, and psychosomatic health status indicators while accounting for potential confounding effects, univariate and multivariate general linear models (GLMs) were applied. In univariate GLM analyses, individual hormonal parameters (cortisol and testosterone and in females, 17-β-estradiol) were included as dependent variables, while stress indicators (PSS, POMS and HBSC categories) were entered as fixed factors and age was included as a covariate. Model assumptions for the general linear models, including normality of residuals and homogeneity of variance, were evaluated using residual diagnostics (including visual inspection of residual plots) and were considered acceptable. In addition to including age as a covariate in the GLM analyses, age-group comparisons were performed to explore developmental differences across the studied population. Analyses were performed separately for males and females.

Multivariate GLM analyses were conducted to assess the combined effect of stress, mood and psychosomatic health status indicators on the hormonal profile. In these models, hormonal parameters were included simultaneously as dependent variables, stress indicators were entered as fixed factors, and age was included as a covariate. Interaction effects between stress indicators were also examined. Multivariate significance was evaluated using Wilks’ Lambda.

Effect sizes were expressed as partial eta squared (partial η^2^), and interpreted according to conventional benchmarks (negligible: −0.009, small: 0.01–0.059, medium: 0.06–0.139, large: 0.14-). Statistical significance was set at *p* < 0.05.

## 3. Results

Due to the non-normal distribution of hormonal variables, non-parametric statistical methods were applied. Significant deviations from normality were confirmed by the Shapiro–Wilk test (males: testosterone *p* = 0.046 and cortisol *p* = 0.003; females: testosterone *p* < 0.001, cortisol *p* < 0.001, and estradiol *p* < 0.001). Continuous variables are presented as median, interquartile range (IQR), and range (Table 2, Table 3 and Table 4).

Age-related differences in hormonal levels were assessed using the Kruskal–Wallis test. No significant differences were observed in testosterone levels across age groups in males (*p* = 0.261, η^2^ = 0.004—negligible size effect). In contrast, cortisol levels in males (*p* = 0.042, η^2^ = 0.021—small size effect) and all hormonal parameters in females (testosterone *p* = 0.003, η^2^ = 0.041—small size effect; cortisol *p* < 0.001, η^2^ = 0.061—large size effect; estradiol *p* < 0.001, η^2^ = 0.141—large size effect) differed significantly across age groups, showing increasing trends with age.

Stress, mood, and psychosomatic indicators were also non-normally distributed (Table 5, Table 6 and Table 7; Shapiro–Wilk test: males—Perceived Stress Scale *p* = 0.014, Profile of Mood States *p* < 0.001, Health Behavior in School-aged Children Symptom Checklist *p* < 0.001; females—Perceived Stress Scale *p* = 0.035, Profile of Mood States *p* < 0.001, Health Behavior in School-aged Children Symptom Checklist *p* < 0.001) and were therefore analyzed using the Kruskal–Wallis test. All indicators showed significant age-related differences, with decreasing trends observed in both males and females (all *p* < 0.001—males: Perceived Stress Scale—η^2^ = 0.020—small size effect; Profile of Mood States—η^2^ = 0.033—small size effect; Health Behavior in School-aged Children Symptom Checklist—η^2^ = 0.014—small size effect, females: Perceived Stress Scale—η^2^ = 0.034—small size effect; Profile of Mood States—η^2^ = 0.101—medium size effect; Health Behavior in School-aged Children Symptom Checklist—η^2^ = 0.081—medium size effect).

Comparisons of hormonal parameters across stress, mood, and psychosomatic health status categories (Table 8) revealed no statistically significant differences in either sex (Kruskal–Wallis test; perceived stress categories—testosterone: males *p* = 0.338, η^2^ = 0.002—negligible size effect, females *p* = 0.196—η^2^ = 0.001—negligible size effect; estradiol: females *p* = 0.439—η^2^ = 0.001—negligible size effect; cortisol: males *p* = 0.501—η^2^ = 0.001—negligible size effect, females *p* = 0.277—η^2^ = 0.002—negligible size effect; mood state categories—testosterone: males *p* = 0.338, η^2^ = 0.002—negligible size effect, females *p* = 0.123—η^2^ = 0.010—small size effect; estradiol: females *p* = 0.691—η^2^ = 0.010—small size effect; cortisol: males *p* = 0.860—η^2^ = 0.001—negligible size effect, females *p* = 0.060—η^2^ = 0.022—medium size effect; psychosomatic health status categories—testosterone: males *p* = 0.668, η^2^ = 0.001—negligible size effect, females *p* = 0.160—η^2^ = 0.006—negligible size effect; estradiol: females *p* = 0.681—η^2^ = 0.001—negligible size effect; cortisol: males *p* = 0.476—η^2^ = 0.004—negligible size effect, females *p* = 0.192—η^2^ = 0.005—negligible size effect).

**Figure 1:**
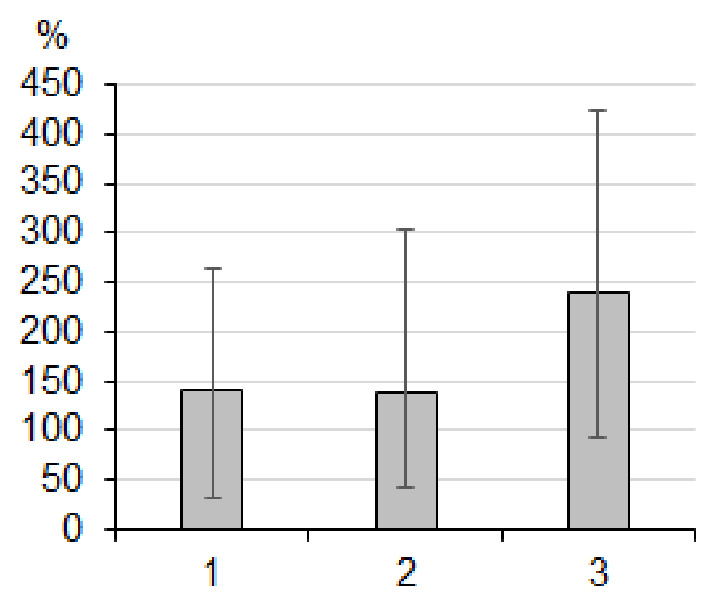
Relative salivary cortisol levels (%) across psychosomatic symptom severity (HBSC-SCL categories) in male handball players—data are presented as estimated marginal means (±95% confidence intervals) and derived from general linear models adjusted for age.

**Figure 2:**
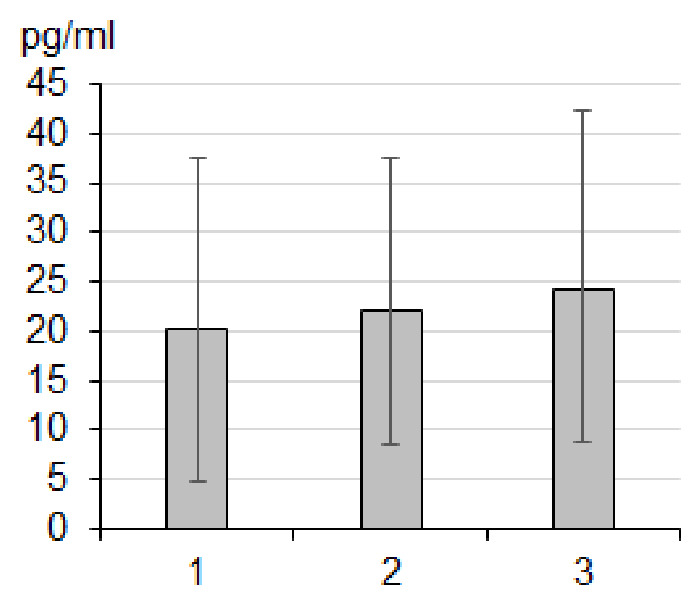
Salivary testosterone levels (pg/mL) across psychosomatic symptom severity (HBSC-SCL categories) in female handball players—data are presented as estimated marginal means (±95% confidence intervals) and derived from general linear models adjusted for age.

The results of the univariate general linear models are presented in Table 9. No significant associations were observed between perceived stress or mood disturbance and hormonal parameters in either sex (all *p* > 0.05). In contrast, psychosomatic symptom severity (HBSC-SCL) showed significant associations with cortisol levels in male athletes (F = 12.43, *p* < 0.001, partial η^2^ = 0.043) and testosterone levels in female athletes (F = 4.78, *p* = 0.009, partial η^2^ = 0.095). Effect sizes were small (partial η^2^ = 0.043) for cortisol in males and medium (partial η^2^ = 0.095) for testosterone in females, indicating limited explanatory power of these associations. As shown in Figure 1 and Figure 2, higher levels of psychosomatic symptoms were associated with increased cortisol levels in males and increased testosterone levels in females. The differences between non-parametric subgroup comparisons and GLM results reflect the adjustment for covariates and the multivariable modeling approach in GLM analyses, which may detect associations not evident in unadjusted comparisons.

Multivariate general linear models revealed a significant overall effect of stress, mood, and psychosomatic indicators on the combined hormonal profile in both males (*p* = 0.048) and females (*p* < 0.001). In male athletes, psychosomatic symptom severity (HBSC) remained significantly associated with the hormonal profile (F = 3.10, *p* = 0.017, partial η^2^ = 0.060, Table 10). In addition, a significant interaction between perceived stress and mood disturbance was observed (PSS × POMS: F = 2.82, *p* = 0.026, partial η^2^ = 0.055), indicating that their combined effect influences hormonal regulation. No significant main effects or interactions were identified in female athletes (all *p* > 0.05).

## 4. Discussion

The present study aimed to investigate the association between endocrine status and stress, mood, and psychosomatic indicators in elite handball players. The main findings indicate that, although both hormonal parameters and stress-related indicators demonstrated clear age-related trends, no consistent associations were observed between endocrine status and perceived stress or mood disturbance. In contrast, psychosomatic symptom severity showed significant relationships with hormonal parameters, particularly cortisol in male athletes and testosterone in females.

The observed age-related patterns are consistent with known developmental and training-related adaptations in elite athletes [24,25]. Hormonal parameters generally increased with age, especially among female athletes, reflecting maturation and cumulative training exposure. In parallel, stress, mood, and psychosomatic indicators showed decreasing trends, suggesting improved coping capacity and adaptation to training demands. These findings highlight that both physiological and psychological systems undergo substantial changes during adolescence and early adulthood in elite sport contexts.

The most important finding of the present study is that psychosomatic symptom severity, assessed using the HBSC checklist, demonstrated the most consistent, albeit modest, association with hormonal parameters. In male athletes, higher levels of psychosomatic symptoms were associated with elevated cortisol levels, while in female athletes, associations were observed with testosterone levels (these results should be interpreted with caution, as cortisol levels may have been influenced by variability in sampling conditions, particularly the lack of strict control over wake-up times). In addition, salivary cortisol levels are known to exhibit substantial inter-individual variability and are highly sensitive to methodological factors, including circadian timing, recent physical activity, and pre-sampling conditions. Previous research has highlighted the importance of controlling these factors when interpreting cortisol measurements in athletic populations [26]. These sources of variability should be considered when interpreting the associations observed in the present study. However, these associations were characterized by relatively small effect sizes, indicating limited explanatory power and suggesting limited practical or clinical relevance. Furthermore, the observed associations may be influenced by unmeasured factors such as training load, recovery status, sleep quality, nutritional status, or competition schedule, which were not assessed in the present study and may contribute to both hormonal variation and psychosomatic symptom reporting. These results suggest that psychosomatic symptoms may reflect underlying physiological strain more closely than subjective perceptions of stress or mood disturbance. While perceived stress and mood states are influenced by cognitive appraisal and contextual factors, psychosomatic complaints represent functional manifestations of stress that may be more directly linked to physiological dysregulation.

This interpretation is supported by previous research indicating that subjective stress perception and physiological stress responses are only weakly related. Associations between perceived stress and cortisol are often inconsistent and generally small, suggesting that these measures capture different aspects of the stress response [27]. In addition, athlete monitoring studies demonstrate that subjective and objective indicators frequently do not correlate, yet provide complementary information [28]. Psychosomatic symptoms, by integrating bodily and perceptual dimensions of stress, may therefore offer a more functionally relevant reflection of an athlete’s physiological state. The results of the present study support this hypothesis, as psychosomatic indicators demonstrated stronger associations with endocrine status than questionnaire-based measures of perceived stress and mood. In line with multidimensional models of stress and recovery, these findings support the concept that combining physiological and functional indicators improves the sensitivity of monitoring approaches in elite athletes.

In contrast, no significant associations were observed between hormonal parameters and perceived stress or mood disturbance. This finding suggests that subjective stress perception and emotional state alone may not adequately reflect physiological strain in elite athletes. High-level athletes are routinely exposed to chronic training demands and competitive stress but often develop effective coping strategies, which may attenuate the relationship between perceived stress and physiological responses. Consequently, perceived stress and mood disturbance may not accurately capture underlying endocrine processes in well-trained populations.

The absence of significant associations between hormonal parameters and perceived stress or mood disturbance is consistent with the heterogeneous findings reported in the literature [10,28,29]. While some studies report weak associations between subjective stress measures and physiological markers such as cortisol, these relationships are typically small and highly variable. In many cases, no significant associations are observed, particularly under resting conditions or in well-adapted athlete populations. These findings suggest that subjective stress perception and endocrine responses represent partially independent components of the stress response, which may not be closely aligned under chronic or habituated conditions.

Our previous analysis [7] demonstrated that endocrine profiles in elite handball players differ substantially from those of non-athlete populations, both in absolute values and distribution patterns. Based on these findings, we initially hypothesized that specific hormonal combinations, such as testosterone–cortisol in males and estradiol–cortisol in females, could serve as indicators of imbalance. While these pairwise approaches provide useful initial insights, the results of the present study suggest that endocrine regulation in athletes is more complex and cannot be fully explained by isolated hormone relationships alone. Instead, the observed associations with psychosomatic symptoms highlight the importance of considering multidimensional interactions between physiological and functional indicators.

The multivariate analyses further support this interpretation, demonstrating that combinations of stress-related factors, rather than single variables, influence hormonal profiles. The observed interaction between perceived stress and mood disturbance indicates that psychological factors may exert combined effects on endocrine responses, even when individual associations are not statistically significant. These findings emphasize the importance of considering complex interactions between psychological and physiological systems in athlete monitoring.

From a practical perspective, these results have important implications for athlete health monitoring and early detection of maladaptation. While hormonal biomarkers provide objective indicators of physiological regulation, psychosomatic symptoms appear to offer complementary and potentially more sensitive information regarding athletes’ functional status. The integration of endocrine and psychosomatic indicators may therefore enhance the identification of athletes at risk of maladaptive responses to training. Previous research has shown that subjective and objective measures often do not correlate but provide complementary information, supporting the use of integrated monitoring approaches [10,28]. In addition, biomarker research emphasizes that combining multiple physiological indicators improves the detection of training stress and overtraining risk. Together, these findings highlight the value of multidimensional monitoring frameworks in elite sport.

In summary, the findings of this study indicate that psychosomatic symptom severity showed statistically significant but modest associations with endocrine status compared to perceived stress or mood disturbance in elite handball players. These results support the use of integrated monitoring approaches that combine physiological and psychosomatic indicators to improve the early detection of maladaptation in elite athletes.

From a practical perspective, the results suggest that routine monitoring of psychosomatic symptoms may provide a simple and sensitive tool for identifying early signs of physiological strain that are not captured by perceived stress or mood measures alone. In applied settings, combining brief psychosomatic questionnaires (e.g., HBSC) with hormonal monitoring may enhance the detection of maladaptive responses in training and support more informed adjustments of training load and recovery strategies. In particular, increases in psychosomatic symptom frequency—even in the absence of elevated perceived stress—may indicate the need for closer monitoring, load modification, or targeted recovery interventions.

### Limitations

The present study has several limitations. The cross-sectional design precludes conclusions regarding causal relationships between psychological indicators and hormonal parameters. Although saliva sampling provides a practical and non-invasive method for assessing endocrine status, variability related to sampling conditions—particularly the lack of strict control over wake-up time, which is a key determinant of cortisol levels due to its pronounced circadian rhythm—may have affected the measurements. In addition, variability in salivary cortisol levels may have been influenced by uncontrolled pre-sampling factors such as recent physical activity, caffeine intake, hydration status, nutritional intake and variability in awakening time. Furthermore, objective measures of training load, sleep quality, and recovery status were not available, although these factors may influence both hormonal responses and psychosomatic symptoms. In female athletes, classification of menstrual cycle phase was based on self-reported data, which may have introduced some degree of misclassification error.

Although several associations reached statistical significance, the observed effect sizes were relatively small, indicating limited explanatory power and practical relevance.

Psychological variables were assessed using self-report questionnaires, which are inherently subject to reporting bias and may not fully capture underlying psychological states. The categorization of psychological variables was based on established cut-offs to facilitate interpretation, although this approach may reduce statistical power and obscure continuous relationships.

The findings reflect relative hormonal variations within a specific population of elite athletes and should therefore not be interpreted as indicators of clinical risk or endocrine dysfunction.

## Figures and Tables

**Table 1:** The distribution of elite handball players by sex and age.

Age (Years)	Males	Females
14	25	61
15	73	49
16	31	39
17	30	38
18	30	19
19	28	31
20–22	47	28
23–35	28	27
Total	292	292

**Table 2:** Descriptive statistics of salivary testosterone concentrations (pg/mL) by sex and age group (N: case number, M: median, interquartile range: P25 and P75—25th and 75th centiles, range: Min: minimum and Max: maximum).

	Males						Females					
	N	M	Min	Max	P25	P75	N	M	Min	Max	P25	P75
U15	95	74.1	14.8	306.0	49.3	116.2	91	17.6	1.8	47.1	10.6	26.9
U17	55	79.9	35.8	199.9	49.3	116.2	43	13.6	1.2	38.3	10.5	18.8
U19	50	89.5	37.5	199.2	61.4	112.4	62	18.4	0.2	90.3	11.8	29.0
U21	59	85.0	41.7	180.5	64.9	121.8	62	21.7	0.2	50.7	16.3	28.4
Adults	33	82.1	41.6	132.2	61.0	100.6	40	21.7	4.9	73.2	12.5	32.5

**Table 3:** Descriptive statistics of salivary estradiol concentrations (pg/mL) by age group in females (N: case number, M: median, interquartile range: P25 and P75—25th and 75th centiles, range: Min: minimum and Max: maximum).

	N	M	Min	Max	P25	P75
U15	91	2.1	0.9	7.3	1.7	3.3
U17	43	3.7	1.4	7.2	2.3	4.0
U19	62	3.3	0.2	8.4	2.8	4.4
U21	62	3.3	2.1	6.9	2.8	4.2
Adults	40	3.0	0.2	5.4	2.5	3.9

**Table 4:** Descriptive statistics of relative salivary cortisol concentrations (%) by sex and age group (N: case number, M: median, interquartile range: P25 and P75—25th and 75th centiles, range: Min: minimum and Max: maximum).

	Males						Females					
	N	M	Min	Max	P25	P75	N	M	Min	Max	P25	P75
U15	95	98.8	16.1	584.3	72.6	174.6	91	90.9	5.5	404.7	50.5	154.3
U17	55	127.0	38.6	570.2	72.6	174.6	43	118.3	24.3	356.0	77.2	184.0
U19	50	104.9	32.0	321.4	85.7	197.9	62	135.2	18.2	472.0	85.4	179.4
U21	59	136.6	50.3	365.7	75.4	143.8	62	142.6	31.4	523.7	100.4	200.9
Adults	33	153.0	44.1	476.6	92.5	193.2	40	95.4	25.5	269.5	71.8	136.8

**Table 5:** Descriptive statistics of Perceived Stress Scale score by sex and age group (N: case number, M: median, relative frequencies of categories—1: low stress level, 2: moderate stress level, 3: high stress level).

	Males					Females				
	N	M	1	2	3	N	M	1	2	3
U15	89	17	56.2	43.8	0.0	81	21	33.3	65.4	1.2
U17	42	16.5	57.1	42.9	0.0	40	19	45.0	55.0	0.0
U19	45	17	53.3	46.7	0.0	59	22	28.8	71.2	0.0
U21	56	17	51.8	48.2	0.0	53	21	30.2	67.9	1.9
Adults	30	11	76.7	23.3	0.0	31	17	58.1	41.9	0.0

**Table 6:** Descriptive statistics of Profile of Mood States score by sex and age group (N: case number, M: median, relative frequencies of categories—1: low mood disturbance, 2: moderate mood disturbance, 3: high mood disturbance).

	Males					Females				
	N	M	1	2	3	N	M	1	2	3
U15	29	8	27.6	44.8	27.6	80	15.5	21.3	51.2	27.5
U17	25	16	24.0	44.0	32.0	21	19	28.6	38.1	33.3
U19	6	1	33.3	66.7	0.0	20	25.5	10.0	50.0	40.0
U21	19	3	31.6	42.1	26.3	16	25	6.3	50.0	43.8
Adults	30	−1	50.0	36.7	0.0	30	−2.5	56.7	30.0	13.3

**Table 7:** Descriptive statistics of Health Behaviour in School-aged Children Symptom Checklist score (HBSC-SCL, 8 items) by sex and age group (N: case number, M: median, relative frequencies of categories—1: rare psychosomatic symptoms, 2: average level of psychosomatic symptoms, 3: frequent psychosomatic symptoms).

	Males					Females				
	N	M	1	2	3	N	M	1	2	3
U15	89	3	89.9	6.7	3.4	81	5	77.8	11.1	11.1
U17	42	4	90.5	4.8	4.8	40	6	72.5	25.0	2.5
U19	45	3	84.4	8.9	6.7	59	7	50.8	39.0	10.2
U21	56	2	87.5	10.7	1.8	53	6	71.7	11.3	17.0
Adults	30	2	96.7	3.3	0.0	31	2	96.8	3.2	0.0

**Table 8:** Median salivary hormone (T: testosterone, E: estradiol, C: cortisol) concentrations across stress, mood, and psychosomatic health status indicators (PSS: Perceived Stress Scale, POMS: Profile of Mood States score, HBSC8: Health Behaviour in School-aged Children Symptom Checklist) categories in male and female handball players.

	PSS			POMS			HBSC8		
	T	E	C	T	E	C	T	E	C
	Males								
1	87.8	–	121.0	73.8	–	134.4	81.0	–	118.2
2	80.8	–	114.9	73.8	–	112.9	83.9	–	103.4
3		–		69.5	–	127.0	102.0	–	137.3
	Females								
1	19.3	3.3	122.7	20.3	2.7	121.2	17.6	3.0	120.7
2	18.0	2.9	112.6	16.3	2.6	95.8	16.7	3.3	101.6
3	37.7	2.9	205.0	16.6	2.7	87.4	22.7	3.0	132.6

**Table 9:** Results of univariate general linear models examining the association between salivary hormonal levels and stress, mood, and psychosomatic health status indicators (PSS: Perceived Stress Scale, POMS, Profile of Mood States score, HBSC8: Health Behaviour in School-aged Children Symptom Checklist).

	F	*p*	Partial η^2^	F	*p*	Partial η^2^
	Males			Females		
Testosterone						
PSS	2.946	0.058	0.013	0.066	0.936	0.101
POMS	1.549	0.217	0.029	1.266	0.285	0.124
HBSC8	0.853	0.427	0.004	4.782	0.009	0.095
Estradiol						
PSS	–	–	–	1.595	0.205	0.025
POMS	–	–	–	0.120	0.887	0.060
HBSC8	–	–	–	0.666	0.515	0.028
Cortisol						
PSS	0.890	0.346	0.012	0.430	0.651	0.008
POMS	1.773	0.175	0.078	0.633	0.533	0.025
HBSC8	12.432	<0.001	0.043	0.779	0.460	0.009

**Table 10:** Results of multivariate general linear models examining the association between salivary hormonal levels and stress, mood, and psychosomatic health status indicators (PSS: Perceived Stress Scale, POMS, Profile of Mood States score, HBSC8: Health Behaviour in School-aged Children Symptom Checklist, *p*: significance level in Wilks lambda test, corrected models were significant: males *p* = 0.048, females *p* < 0.001).

	F	*p*	Partial η^2^	F	*p*	Partial η^2^
	Males			Females		
PSS	0.243	0.785	0.005	0.780	0.586	0.016
POMS	1.496	0.205	0.032	1.448	0.196	0.029
HBSC8	3.103	0.017	0.060	0.647	0.692	0.013
PSS × POMS	2.820	0.026	0.055	0.924	0.431	0.019
PSS × HBSC8	2.817	0.051	0.058	0.440	0.725	0.009
POMS × HBSC8	0.399	0.672	0.008	1.937	0.058	0.039

## Data Availability

The datasets generated during and analyzed during the current study are not publicly available due to personal data of the participants but are available from the corresponding author on reasonable request.
